# Compound heterozygous TYK2 mutations underlie primary immunodeficiency with T-cell lymphopenia

**DOI:** 10.1038/s41598-018-25260-8

**Published:** 2018-05-03

**Authors:** Michiko Nemoto, Hiroyoshi Hattori, Naoko Maeda, Nobuhiro Akita, Hideki Muramatsu, Suzuko Moritani, Tomonori Kawasaki, Masami Maejima, Hirotaka Ode, Atsuko Hachiya, Wataru Sugiura, Yoshiyuki Yokomaku, Keizo Horibe, Yasumasa Iwatani

**Affiliations:** 1 Clinical Research Center, National Hospital Organization Nagoya Medical Center, Aichi, 460-0001 Japan; 2 Graduate School of Environmental and Life Science, Okayama University, Okayama, 700-8530 Japan; 3 Department of Pediatrics, National Hospital Organization Nagoya Medical Center, Aichi, 460-0001 Japan; 4 Department of Pediatrics, Nagoya University Graduate School of Medicine, Aichi, 466-8550 Japan; 5 Department of Pathology, National Hospital Organization Nagoya Medical Center, Aichi, 460-0001 Japan; 6 Division of Basic Medicine, Nagoya University Graduate School of Medicine, Aichi, 466-8550 Japan

## Abstract

Complete tyrosine kinase 2 (TYK2) deficiency has been previously described in patients with primary immunodeficiency diseases. The patients were infected with various pathogens, including mycobacteria and/or viruses, and one of the patients developed hyper-IgE syndrome. A detailed immunological investigation of these patients revealed impaired responses to type I IFN, IL-10, IL-12 and IL-23, which are associated with increased susceptibility to mycobacterial and/or viral infections. Herein, we report a recessive partial TYK2 deficiency in two siblings who presented with T-cell lymphopenia characterized by low naïve CD4^+^ T-cell counts and who developed Epstein-Barr virus (EBV)-associated B-cell lymphoma. Targeted exome-sequencing of the siblings’ genomes demonstrated that both patients carried novel compound heterozygous mutations (c.209_212delGCTT/c.691C > T, p.Cys70Serfs*21/p.Arg231Trp) in the *TYK2*. The *TYK2* protein levels were reduced by 35% in the T cells of the patient. Unlike the response under complete TYK2 deficiency, the patient’s T cells responded normally to type I IFN, IL-6, IL-10 and IL-12, whereas the cells displayed an impaired response to IL-23. Furthermore, the level of STAT1 was low in the cells of the patient. These studies reveal a new clinical entity of a primary immunodeficiency with T-cell lymphopenia that is associated with compound heterozygous *TYK2* mutations in the patients.

## Introduction

Interferons (IFN) and other cytokines, which play important roles in multiple innate and adaptive immune responses, transduce signals via the JAK-STAT pathway. When the cytokines bind and induce the dimerization of their receptors, receptor-associated Janus kinases (JAKs) become phosphorylated and activated. The activated JAKs then phosphorylate downstream substrates, the signal transducers and activators of transcription (STAT) molecules, which subsequently dimerize and translocate to the nucleus to activate the transcription of specific genes. Mutations of the genes encoding components of the JAK-STAT pathway cause various immunological disorders, including increased susceptibility to infection, such as in growth hormone insensitivity syndrome, severe combined immunodeficiency, and others^1–11^.

One of the JAKs, tyrosine kinase 2 (TYK2), which is associated with the receptors of type I IFN, interleukin (IL)-6, IL-10, IL-12 and IL-23, plays a central role in the signal transduction of these cytokines^12,13^. TYK2 deficiency was first described in a 22-year-old Japanese male patient who developed symptoms of hyper-IgE syndrome (HIES) with susceptibility to various pathogens, including *Staphylococcus*, mycobacteria and herpes simplex virus^2^. Genomic DNA sequencing analysis in the patient has revealed a homozygous frame-shift mutation in the *TYK2* gene, which resulted in a frameshift at codon 90 with the premature termination of translation. Therefore, the patient’s cells expressed no functional TYK2 protein that could be detected via immunoblot analysis. The cells derived from the TYK2-deficient patient displayed nearly abolished responses to type I IFN, IL-12, IL-23, IL-6 and IL-10. More recently, the comprehensive immunological investigation of seven other TYK2-deficient patients has been reported^14^. Unlike the first TYK2-deficient patient, cells from these TYK2-deficient patients displayed an impaired but not abolished response to type I IFN, IL-12, IL-23 and IL-10. The study suggested that the susceptibility to intracellular bacterial and/or viral infections identified in all the TYK2-deficient patients was caused by impaired responses to IL-12 and type I IFN^14^. All of these accumulating reports have elucidated the functional impacts of a complete TYK2-deficiency on clinical outcomes. However, little is known regarding the functional impact of other *TYK2* variants (e.g., insertion, deletion and substitution).

In this study, we present two cases of patients who had immunodeficiency associated with novel heterozygous mutations in the four-point-one, ezrin, radixin, moesin (FERM) domain region of *TYK2*. Unlike previous reports on TYK2 deficiency, the patients exhibited severe T-cell lymphopenia characterized by low naïve CD4^+^ T-cell counts. Furthermore, both patients developed Epstein-Barr virus (EBV)-associated B-cell lymphoma. We analyzed the responses to cytokines in the cells of the patient. The cellular functions partially resemble but are less defective than those previously described in the TYK2-deficient patient.

## Results

### Characteristics of Patients

**Figure 1:**
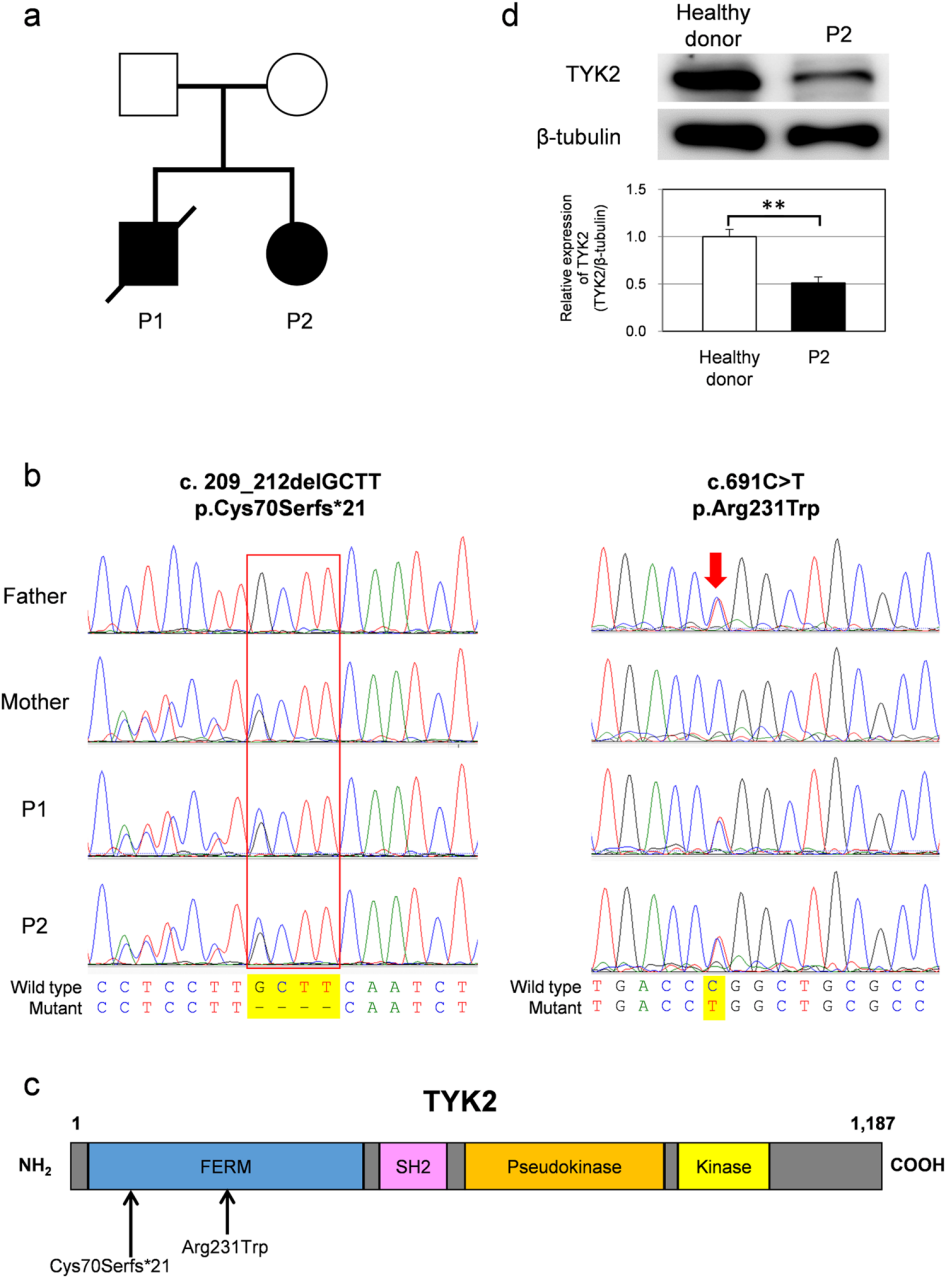
Identification of *TYK2* compound heterozygous mutations in siblings with primary immunodeficiency. (**a**) Pedigree of a family in which compound heterozygous mutations in *TYK2* were identified. Squares and circles denote males and females, respectively. Closed boxes indicate affected individuals, and a diagonal bar represents a deceased individual. (**b**) Validation by Sanger sequencing of the *TYK2* mutations in the patients and their parents. (**c**) Schematic representation of the TYK2 protein. (**d**) western blot analysis of TYK2 protein expression in EBV-BCLs established from the PBMCs of a healthy donor and the *TYK2*-mutated patient P2. ß-tubulin served as a loading control. n = 3 EBV-BCLs per genotype. Data represent mean ± SEM. **P < 0.01. P values were derived from 2-tailed Student’s t-test. Full-length immunoblots are presented in Supplementary Fig. S6.

**Figure 2:**
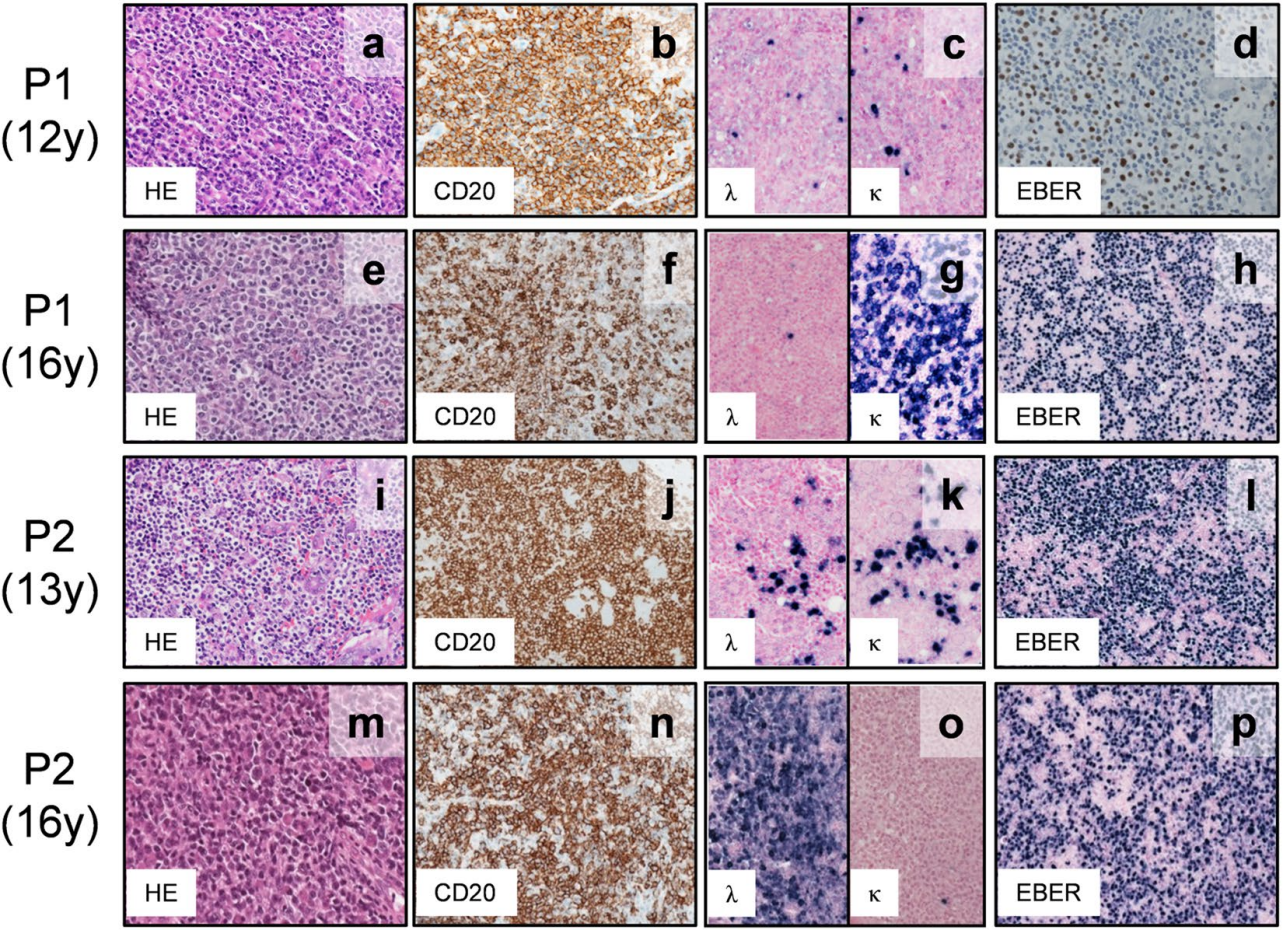
Histologic analysis of biopsy specimens from the patients. A biopsy specimen from the parotid gland of P1 at 12 years of age (**a–d**) and at 16 years of age (**e–h**). The parotid gland of P2 at 13 years of age (**i–l**); the left nasopharynx of P2 at 16 years of age (**m–p**). Staining with hematoxylin and eosin (HE) and immunostaining with anti-CD20 antibody demonstrates the infiltration of CD20^+^ lymphocytes. An *in situ* hybridization study demonstrated EBV-encoded RNAs (EBERs). The cell clonality was assessed by *in situ* hybridization for *κ* and *λ* mRNAs.

Patient 1 (P1) was referred as a 15-year-old boy born to non-consanguineous parents (Fig. 1a). He had a history of varicella and recurrent parotitis. He had received the following vaccinations: bacille Calmette-Guérin (BCG); diphtheria-pertussis-tetanus (DPT); polio; influenza; measles-rubella (MR) and Japanese encephalitis. A parotid biopsy was performed at 12 years of age and exhibited diffuse medium- to large-sized B-cell proliferation (Fig. 2a–c). Furthermore, a lymphoepithelial lesion was detected (Fig. 2a). The lesion was diagnosed as mucosa-associated lymphoid tissue (MALT)-type B-cell lymphoma (stage III). He was treated with chemotherapy according to the reference standard, and he initially obtained remission. At the age of 16 years, EBV-associated diffuse large B-cell lymphoma (stage III) was diagnosed by parotid gland biopsy. Large B cells were expanded monoclonally (Fig. 2e–g). These cells as well as a biopsy sample at the age of 12 years were positive for EBV-encoded RNAs (EBERs) (Fig. 2d,h). Positivity for anti-EBV viral capsid antigen (VCA) IgG was observed without anti-VCA IgM antibodies, and the EBV DNA copy number in the peripheral blood was 5,200 copies/mL (Table 1). The patient died at the age of 16 years due to a severe graft-versus-host disease (GVHD) after allogeneic bone marrow transplantation (BMT).

**Table 1:** Detection of EBV infection.

Characteristic	Patient 1 (P1)	Patient 2 (P2)
Serology^A^		
Anti-VCA IgG	1:2,560	1:640
Anti-VCA IgM	<1:10	<1:10
Anti-EA-DR IgG	Negative	1:20
Anti-EBNA IgG	<1:10	1:80
Viral load (copies/mL)	5,200	4,467
EBER probe	Positive	Positive

^A^Antibody titers.

VCA, viral capsid antigen; EA-DR, early antigen-diffuse and restricted; EBNA, EBV nuclear antigen; EBER, EBV-encoded RNA.

Patient 2 (P2), the 14-year-old sister of P1 exhibited similar symptoms. She had a history of varicella and recurrent otitis media with effusion. She had received vaccines of BCG, DPT, polio, influenza and MR. At the age of 13 years, a biopsy of the right parotid gland was histologically diagnosed as EBV-associated B-cell lymphoma (stage II). The biopsy exhibited a diffuse lymphoid infiltrate composed of small to medium lymphocytes. The lymphoid cells included monocytoid B cells and centrocyte-like cells, reminiscent of MALT-type B-cell lymphoma (Fig. 2i–k). The cells were positive for EBERs (Fig. 2l). P2 received chemotherapy and achieved provisional remission. At the age of 16 years, she developed EBV-associated B-cell lymphoma (stage II) in the left nasopharynx (Fig. 2m–p). Immunostaining for light chains revealed predominant staining of the κ light chain, indicating monoclonal proliferation of the tumor cells (Fig. 2o). Similar to P1, the patient was positive for anti-EBV VCA IgG in the absence of anti-VCA IgM antibodies. A PCR analysis revealed EBV infection with a viral load of 4,467 copies/mL in the peripheral blood (Table 1). P2 underwent another round of chemotherapy and obtained remission. Currently, P2 is being followed up in Japan and exhibited persistent increase in EBV viral load (1.4 × 10^4^ copies/mL) even 15 months after the final chemotherapy. The results of serologic screening for HIV and PCR screening for cytomegalovirus and human herpes virus-6 were negative in both patients.

Immunophenotyping of the peripheral blood mononuclear cells (PBMCs) revealed that both patients had significant T-cell lymphopenia characterized by low naïve CD4^+^ T-cell (CD4^+^CD31^+^CD45RA^+^, recent thymic emigrant cells) counts (Table 2). The frequencies of B cells and NK cells were close to those of age-matched controls. High EBV antibody titers indicated intact B-cell function in the patients (Table 1). The NK cell function could not be successfully evaluated because of inadequate cellular materials. Both patients had impaired T-cell proliferative responses following phytohemagglutinin(PHA) stimulation (Table 2). The immunoglobulin levels in both patients’ sera were normal with the exception of elevated IgA and slightly reduced IgE levels in P2.

**Table 2:** Immunological features of patients P1 and P2.

	Patient 1 (P1)	Patient 2 (P2)	Control adult
Age at evaluation	15y	14y	—
White blood cells (4,400–8,100 cells/μL)^A^	3,100	4,700	—
Lymphocyte (1,400–3,300 cells/μL)^A^	434	658	1,500
CD3^+^ T cell (1,000–2,200 cells/μL)^A^	287	365	1,216
CD4^+^ T cell (530–1,300 cells/μL)^A^	107	178	669
CD8^+^ T cell (330–920 cells/μL)^A^	130	117	416
Memory helper T cell (CD3^+^CD4^+^CD45RO^+^) (240–700 cells/μL)^A^	103	201	338
Recent thymic emigrants (CD4^+^CD31^+^CD45RA^+^) (150–1,500 cells/μL)^B^	14	9	386
Treg (CD4^+^CD25^+^CD127^−^) (33–190 cells/μL)^B^	9	17	49
TCR α/ß (700–2,800 cells/μL)^B^	212	279	1,150
TCR γ/δ (39–540 cells/μL)^B^	32	31	23
NK cell (CD16^+^/CD56^+^) (70–480 cells/μL)^A^	76	50	73
B cell (CD19^+^) (110–570 cells/μL)^A^	59	157	187
Memory B cell (CD19^+^CD27^+^IgD^−^) (12–69 cells/μL) ^C^	3	10	28
T cell proliferation (×10^−3^ cpm)			
PHA (>50)	11.4	5.4	—
Ig levels^D^			
IgM (0.55–1.77 mg/mL)	0.33	1.03	—
IgG (4.8–14.0 mg/mL)	10.4	11.1	—
IgA (0.49–1.90 mg/mL)	2.79	5.22	—
IgE (10–100 U/mL)	23^E^	5^F^	—

^A^Age-matched normal values for lymphocyte counts according to Journal of Allergy and Clinical Immunol 2003; 112:973-80^49^, ^B^Scandinavian Journal of Immunology 2012; 75:436-44^50^, ^C^Immunity, Inflammation and Disease 2014; 2:131-40^51^, ^D^Blood 2012; 119:3458-68^26^. ^E^Analyzed at 16 years of age. ^F^Analyzed at 16 years of age.

To evaluate the lymphocyte proliferation response in P2, T cells were stimulated with anti-CD3 monoclonal antibody (mAb) plus anti-CD28 mAb-coated beads and expanded in culture with IL-2. The result showed that normal T-cell proliferation was restored in P2 compared with the cells derived from her parents and a control donor (Supplementary Fig. S1).

### Identification of causative mutations

Initially, an autosomal recessive inheritance of primary immunodeficiency was suspected because the parents were non-consanguineous and had no symptoms related to immune disorders. Therefore, we performed targeted exome sequencing for P1, P2 and their parents to identify causative or candidate genes. In total, 7 Mbp of exome sequences (targeting approximately 2,761 genes registered as disease-implicated in the Human Genome Mutation Database) were enriched and analyzed with the Illumina MiSeq. The sequencing resulted in 1.1–1.3 Gbp of raw sequence data per patient. In total, 87.8–90.0% of the targeted bases were sequenced with at least 20-fold coverage depth. A genetic variant analysis resulted in the detection of 4,816 (P1) and 4,829 (P2) variants. These variants were further filtered using the following criteria: (1) linked to missense, frameshift, loss or gain of a stop codon, initiator codon, in-frame insertion/deletion, or splice-site mutations; (2) detected as uncommon variants (a frequency of less than 5%, which was set according to previous reports)^15,16^ in the Asian population; (3) inherited in an autosomal recessive pattern; (4) shared by both of the patients. The results revealed that 16 variants in four genes, *TYK2*, *ANK2*, *RYR1* and *DSPP* fulfilled the above criteria (Supplementary Table S1). Of these variants, seven synonymous and four non-synonymous mutations were identified in two genes, *RYR1* and *DSPP*. As biological functions of the two genes are likely irrelevant to primary immunodeficiency, we narrowed down to five non-synonymous variants in *TYK2* and *ANK2* to identify the causative mutations. Of note, our analysis of structural variants (SVs) linked to the T-cell lymphopenia displayed 22 rare SVs that are observed with the expected frequency of less than 5% in either the 1000 Genome Project data or the Exome Aggregation Consortium (ExAC) data^17,18^. However, any of these SVs were not associated with a recessive inheritance (Supplementary Table S2).

In addition, since our patients developed aggressive EBV-associated B-cell lymphoma that is rarely observed in children, we further validated exome sequencing data for the genes of which mutations have been reported in the patients suffering from EBV-associated lymphoproliferative diseases, including EBV-associated lymphoma; IL-2 inducible tyrosine kinase (ITK)^19^, CD27^20^, SH2 domain protein 1A (SH2D1A)^21^, X-linked inhibitor-of-apoptosis protein (XIAP)^22^, Wiskott-Aldrich syndrome protein (WASP)^23^, coronin, actin-binding protein 1A (CORO1A)^24,25^, mammalian sterile 20-like kinase-1 (MST1)^26^ and magnesium transporter 1 (MAGT1)^27^. All of these genes are involved in the survival and/or differentiation of T cells. The results showed that no nonsense mutations were detected in these genes. Nevertheless, further investigations are required for verifying an alternative possibility that unknown other genetic mutations might be associated with aggressive development of EBV-associated B-cell lymphoma under low TYK2 activity.

Our verification of the five mutations by Sanger sequencing indicated that two variants of the *TYK2* gene were validated whereas three mutations of the *ANK2* were false positive on the MiSeq analysis. As shown in Fig. 1b, both parents are heterozygous for the *TYK2* gene localized on chromosome 19: c.209_212 del GCTT in the mother and c.691C > T in the father. Their children, the two patients, carry compound heterozygous mutations in *TYK2* (Fig. 1b). The first *TYK2* mutation, c.209_212 deletion, resulted in a frame-shift mutation and generated a premature stop codon at amino acid residue 90. The homozygous form of this mutation was reported previously^2^ and registered in the dbSNP database (rsID: rs770927552) with a minor allele frequency of 0.00006. The second *TYK2* mutation is a c.691C > T transition and resulted in a p.Arg231Trp substitution in the FERM domain region (Fig. 1c). This transition mutation has been registered in the dbSNP database (rsID: rs201917359) with a minor allele frequency of 0.0008.

These results suggest that the causative mutations are strongly linked to the compound heterozygous mutations, namely the c.209_212 del GCTT and the c.691C > T in the *TYK2* genes of these two patients.

### Assessment of type I IFN response

To probe possible association between the *TYK2* variants and cellular functions, we measured the *TYK2* mRNA and protein levels in EBV-transformed human B-cell lines (EBV-BCLs) prepared from PBMCs of healthy donors and P2. The result indicated that no significant reduction of the mRNA level was observed in P2 compared with healthy donor controls (Supplementary Fig. S2). In contrast, the immunoblot analysis showed that the TYK2 protein level (Fig. 1d, Supplementary Fig. S2) was low in P2 (47–51% relative to the control donors). The reduction was similarly observed in primary CD4^+^ T cells derived from P2, whereas the TYK2 protein levels in CD4^+^ T cells from her parents were similar to the levels in the healthy control (Supplementary Fig. S3). These results suggest no significant impact of single allelic variation for the c.209_212 del GCTT or the c.691C > T on the TYK2 expression.

**Figure 3:**
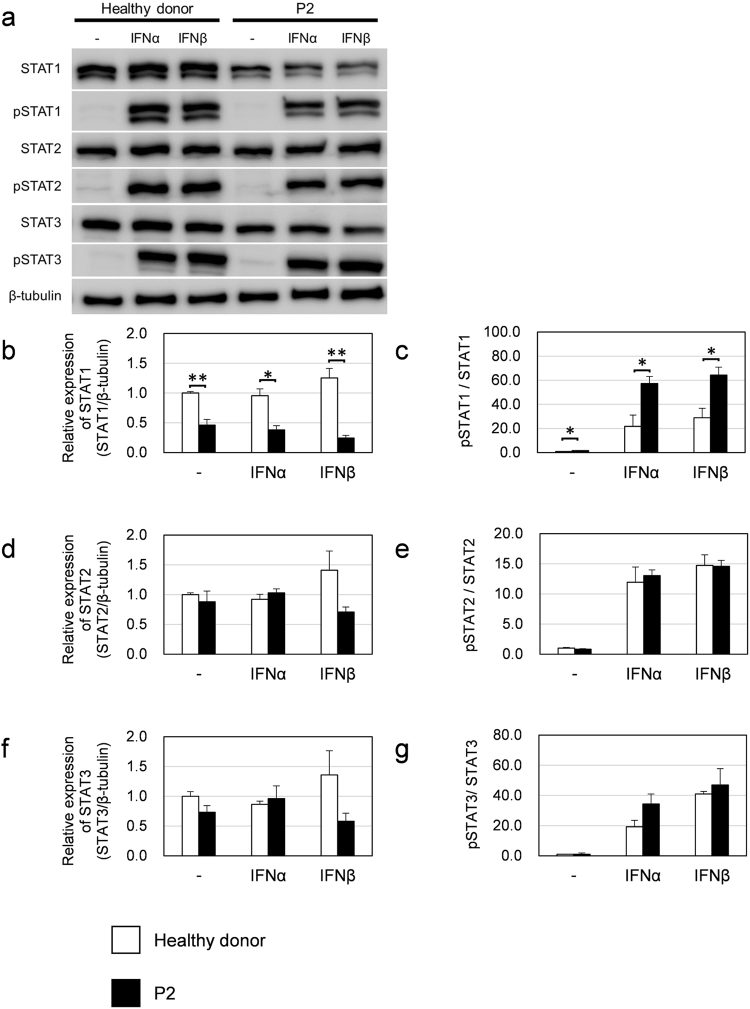
Type I IFN-induced STAT activation in EBV-BCLs from a healthy donor and the patient. (**a**) Total STAT and tyrosine-phosphorylated STAT (pSTAT) protein levels were analyzed by western blotting. Cells were stimulated with IFN-α (1000 U/mL) or IFN-ß (500 U/mL) for 15 min. (**b**–**g**) Based on three independent analyses, the expression levels of STAT proteins (**b,d,f**) and pSTAT proteins (**c,e,g**) in the patient-derived EBV-BCLs were compared with those in untreated healthy donor-derived cells (n = 3 EBV-BCLs per genotype). Data represent mean ± SEM. **P < 0.01, *P < 0.05. P values were derived from 2-tailed Student’s t-test. Full-length immunoblots are presented in Supplementary Fig. S7.

Next, to assess the function of TYK2 in the patient, the levels of STAT1 (Tyr 701), STAT2 (Tyr 690) and STAT3 (Tyr 705) phosphorylation in patient-derived EBV-BCLs and T cells upon stimulation with IFN-α and IFN-ß were analyzed by immunoblotting (Fig. 3a) and compared by quantifying the gel images (Fig. 3c,e and g). The relative expression level of STAT1 was less than 50% with or without IFN stimulation in the patient-derived cells compared with the control cells (Fig. 3b). However, the ratio of phosphorylated STAT1 (pSTAT1) to STAT1 was 2-fold higher in the P2-derived cells than in the control cells, suggesting that the patient-derived cells responded to type I IFN normally or even better than the healthy donor cells (Fig. 3c). In contrast, the relative expression levels of STAT2 (Fig. 3d) and the IFN-induced phosphorylated STAT2 (Fig. 3e) were similar between the P2 cells and the control cells. The levels of STAT3 exhibited a pattern similar to that of STAT2 (Fig. 3f,g).

**Figure 4:**
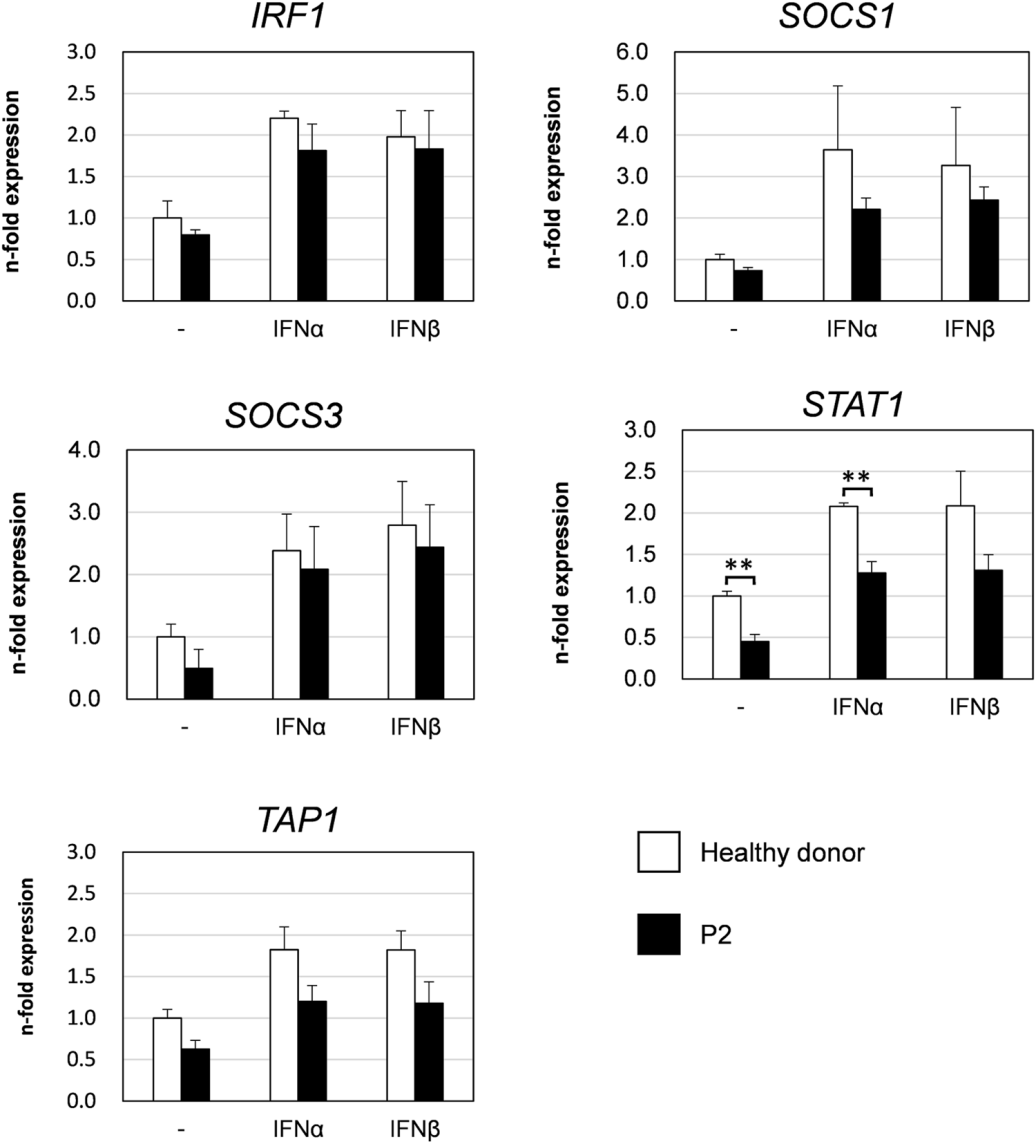
Analysis of type I IFN-inducible gene induction in EBV-BCLs from a healthy donor and the patient.Cells were stimulated with IFN-α (1000 U/mL), IFN-ß (500 U/mL) or without any IFN for 2 h. The cDNAs generated from the total RNA were quantified with qPCR assays. The expression levels of *IRF1*, *SOCS1*, *SOCS3*, *STAT1* and *TAP1* mRNAs were determined by normalizing each with *cyclophilin B* levels. The induction level is presented as n-fold expression that in the untreated healthy donor cell control, which was set as 1. The data are derived from three independent experiments using 3 EBV-BCLs per genotype. Data represent mean ± SEM. **P < 0.01. P values were derived from 2-tailed Student’s t-test.

Furthermore, the transcriptional upregulation of type I IFN-inducible genes, including *IRF1*, *SOCS1*, *SOCS3*, *STAT1* and *TAP1*, were analyzed. One of the JAK-STAT pathway components, STAT1, is itself an IFN-inducible gene product^28^. The transcriptional upregulation of type I IFN-inducible genes in response to type I IFN was comparable to that of the control cells (Fig. 4 and Supplementary Fig. S4). Among the five genes, only low *STAT1* expression significantly differed between the patient’s and healthy donor cells (Fig. 4). These results suggest that the *TYK2* compound heterozygous mutations are associated with a low level of constitutive *STAT1* mRNA expression.

### Assessment of response to IL-6, IL-10, IL-12 and IL-23

**Figure 5:**
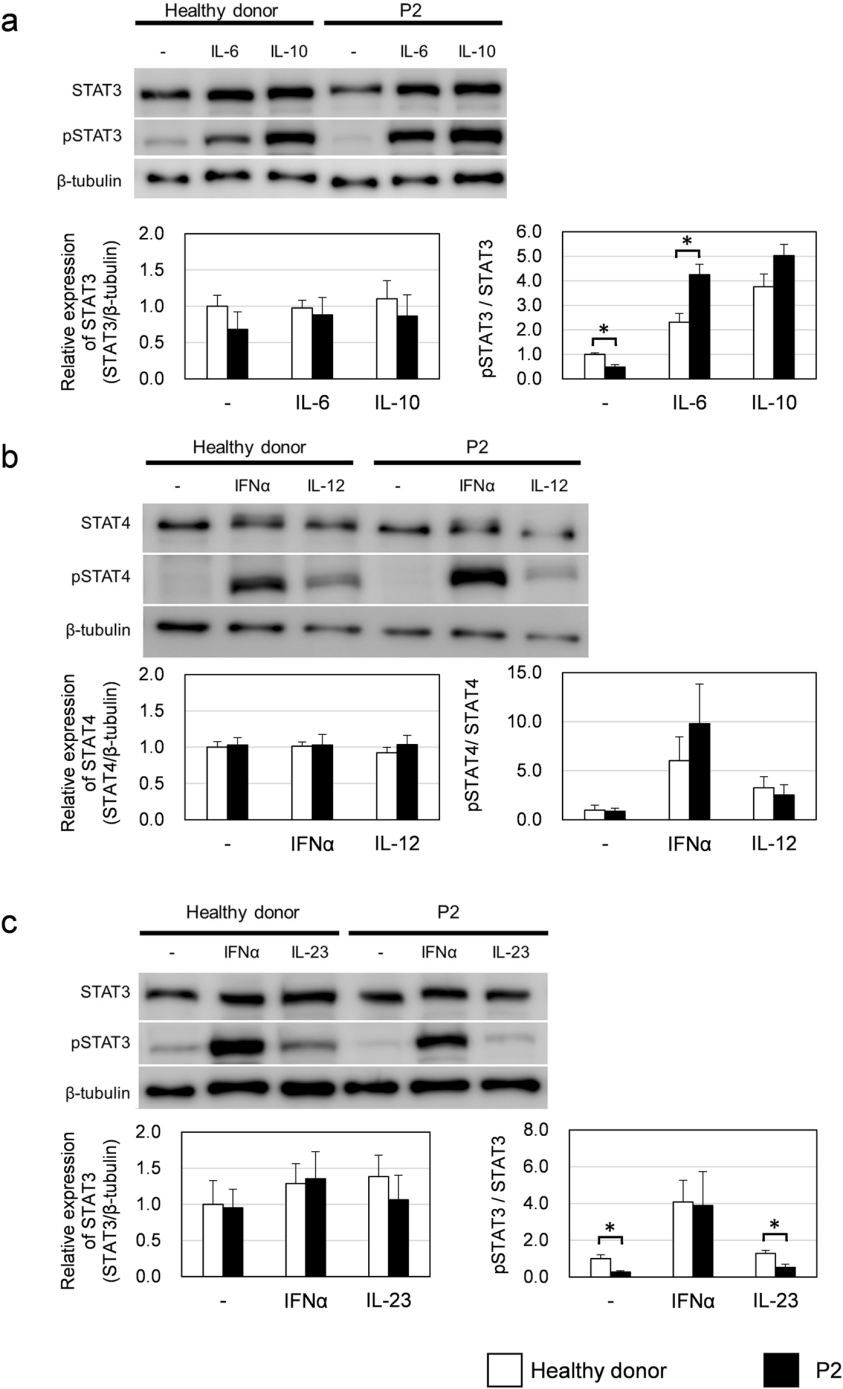
STAT activation in response to IL-6, IL-10, IL-12 and IL-23 in T cells from a healthy donor and the patient.Total STAT and tyrosine-phosphorylated STAT (pSTAT) protein levels were analyzed by western blotting. (**a**) The cells were stimulated with IL-6 (10 ng/mL) or IL-10 (10 ng/mL) for 15 min. (**b**) The cells were stimulated with IL-12 (10 U/mL) or IFN-α (1000 U/mL) for 15 min. (**c**) The cells were stimulated with IL-23 (500 ng/mL) or IFN-α (1000 U/mL) for 15 min. Full-length immunoblots are presented in Supplementary Fig. S8–10. Based on three independent analyses using same batch of CD4^+^ T cells, STAT proteins and pSTAT proteins expression levels were calculated relative to those of untreated healthy donor-derived cells. Data represent mean ± SEM. *P < 0.05. P values were derived from 2-tailed Student’s t-test.

In addition to the type I IFN signaling pathway, TYK2 is also involved in the IL-6 and IL-10 signaling pathways, which regulate a broad range of physiological responses through the activation of STAT3. To assess the IL-6 and IL-10 signaling pathways in the patient, the levels of phosphorylated STAT3 (Tyr 705) proteins in the patient-derived T cells stimulated with IL-6 or IL-10 were analyzed. As depicted in Fig. 5a, the patient’s cells responded similarly or even more efficiently to IL-6 and IL-10 compared to the control cells.

Finally, we tested the effects of the *TYK2* variants on the IL-12 and IL-23 signaling pathways because previous studies using TYK2-deficient cells from an immunodeficient patient or *TYK2*-knockout mice have shown that a lack of TYK2 affects the response to IL-12 and IL-23^2,14,29,30^. IL-12 and IL-23 are pro-inflammatory cytokines that mediate signaling via STAT4 and STAT3, respectively. A comparative analysis of STAT4 phosphorylation (Tyr 693) after IL-12 stimulation revealed a slight reduction in phosphorylation in the P2 cells compared with the control cells (Fig. 5b); however, the difference was not significant. In contrast, the IL-23-dependent phosphorylation of STAT3 (Tyr 705) was reduced in the P2-derived cells (41%) relative to that in the control cells (Fig. 5c). These observations demonstrated that the signaling pathway of IL-23 but not that of IL-12 was significantly impaired in the patient’s cells.

## Discussion

The present study investigated two patients with primary immunodeficiency who developed a type of EBV-associated B-cell lymphoma that is rare in children. Through the targeted exome sequencing analysis of the patients’ genomic DNA, we identified compound heterozygous mutations (c.209_212 del GCTT /c.691 C > T, p.Cys70Serfs*21/p.Arg231Trp) in the TYK2-encoding gene. The four-base deletion locus of the *TYK2* gene (c.209_212 del GCTT) was previously reported in the first TYK2-deficient patient^2^. The missense mutation (c.691 C > T), which causes an Arg231Trp mutation, was previously registered in the dbSNP database; however, the mutational effect was unknown.

Our immunoblot analysis revealed that the patient-derived cells expressed nearly 50% (47–51%) of the TYK2 protein compared with healthy donor cells (Fig. 1d and Supplementary Fig. S2). In contrast, cells isolated from the parents carrying either of the *TYK2* mutations (c.209_212 del GCTT or c.691 C > T) exhibited a level of TYK2 protein similar to that of the cells with wild-type *TYK2* alleles. These data indicated that these *TYK2* mutations might affect the TYK2 protein expression level, and the mutations in the parent’s cells may be compensated by the wild-type allele. The levels of *TYK2* mRNA in the patient were similar to those in the healthy donors (Supplementary Fig. S2). Interestingly, the second mutation (c.691 C > T) that we identified in this study results in the substitution of the Arg231 within the FERM domain of TYK2 for a tryptophan. Mapping of the residue in the domain structure (PDB 4PO6) showed that it is located at positively charged TYK2 surface that is critical for maintaining the FERM F2 conformation stability as well as interacting with plasma/nuclear membrane (Supplementary Fig. S5)^31^. Therefore, the missense mutation (c.691 C > T) may lead to disruption of intracellular localization and/or intracellular stability of TYK2 protein. This mechanistic scenario is strongly supported by a previous finding that alanine substitutions of Arg231-233-235 impaired TYK2 nuclear localization^32^.

The first Japanese patient with TYK2 deficiency reported by Minegishi *et al*.^2^ was characterized by atopic dermatitis with highly elevated serum IgE (2100 IU/mL) and diagnosed with HIES. In contrast, serum IgE concentrations of 23 IU/mL and 5 IU/mL for P1 (at the age of 16 years) and P2 (at the age of 11 years), respectively, were noted in this study; these levels were even lower than those for the healthy control. More recently, another report of seven TYK2-deficient patients has shown that the core clinical phenotype of TYK2 deficiency is due to mycobacterial and/or viral infections and not to HIES^14^.

In addition, the immunological investigation of T cells derived from these patients displayed impaired responses to IFN-α/ß, IL-12, IL-23 and IL-10^14^. Upon IFN-α/ß stimulation, a lower level of STAT1 phosphorylation and no STAT3 phosphorylation was detected in the TYK2-deficient patients^14^. In contrast, our patient-derived cells displayed similar levels of phosphorylated STAT1 and STAT3 proteins in response to IFN-α/ß compared with the TYK2-proficient control cells. Additionally, the transcriptional induction of type I IFN-inducible genes in response to IFN-α/ß was normal in the patient’s PBMCs. The reduced expression of only *STAT1* mRNA and protein was observed in the patient’s cells compared to the control cells. Such STAT1 protein reduction has also been observed in the complete TYK2-deficient patients and in *TYK2*^*-/-*^ mice as previously reported^2,14,29^. Because basal expression levels of IFN-inducible genes, including *STAT1*, are maintained by low amounts of constitutively secreted IFN-ß in immune cells^28,33^, this minimal constitutive activation of STAT signaling might enable immune cells to maintain a rapid immune response to infections. Therefore, the reduced STAT1 expression observed in this study could be attributed to the absence or decreased basal level of TYK2 protein expression that regulates the IFN signaling pathway. Previous reports have suggested apparently inconsistent mechanisms in immunodeficient patients with low levels of STAT1 expression caused by an exonic splicing enhancer mutation in *STAT*1^4,5^ or by insufficiently stable STAT1 protein folding^6^. Consequently, those patients exhibited susceptibility to mycobacterial and viral infections and/or displayed progressive losses in lymphocyte numbers and functions. Further studies are warranted to better understand whether the clinical features of the patients in the present study can be attributed to the effects of reduced STAT1 levels or other unidentified factors associated with TYK2 mutation.

In contrast to the aforementioned immunological features that are in common between our patient and the previously reported patients with complete TYK2 deficiency, there are two immunological signatures that are different. First, STAT3 activation is noted upon IL-6/IL-10 stimulation. Kreins *et al*. have shown that STAT3 phosphorylation after IL-10 stimulation was impaired in cells from complete TYK2-deficient patients, whereas STAT3 phosphorylation upon IL-6 stimulation was normal in those cells, suggesting that TYK2 is indispensable for IL-10 signaling but not for IL-6 signaling^14^. However, we observed no significant impairment in the response to either IL-10 or IL-6 in our patient’s T cells. Residual TYK2 activity might account for the normal IL-6/IL-10 signaling in our patient. Second, approximately normal responses to IL-12 were observed in our patient-derived cells regarding STAT4 phosphorylation, whereas a drastic impairment of IL-12 signaling was detected in the complete TYK2-deficient patients^2,14^. Disorders involving IL-12-dependent signal transduction may result in mycobacterial diseases, such as BCG infection or Salmonella infection^34^. Indeed, BCG and Salmonella infections that are likely due to impaired IL-12 responses have been previously observed in TYK2-deficient patients^2,3,14^. However, the patients in our present study did not have any episodes of BCG or Salmonella infection. This clinical evidence strongly indicates that residual TYK2 activity involving the IL-12 signaling pathway was sufficiently maintained to induce protective immunity against those bacteria.

An intriguing observation of this study was that the patients presented atypical clinical features that have not been observed in TYK2-deficient patients. In addition, as a prominent clinical feature of our patients, T-cell lymphopenia that is characterized by low naïve CD4^+^ T-cell counts was similarly observed in our two patients. The reduction in the T-cell count observed in our patients was not found in previous reports of TYK2-deficient patients or *TYK2*-deficient mice^2,3,14,29,30^. There are several reports that mutations in a variety of genes cause primary immunodeficiency associated with T-cell lymphopenia^6,24,26,35,36^ and that these T-cell lymphopenias are attributed mainly to an impaired proliferative capacity of T cells^26,35,36^ or a high expression of apoptosis-related proteins^6,26^. In the present study, we obtained inconsistent results for the proliferative capacity of T cells in the patient. The proliferative capacity of PBMCs after PHA stimulation were impaired (Table 2), whereas T cells isolated from the patients proliferated in response to anti-CD3 and anti-CD28 antibodies and exhibited no apparent signs of apoptosis (Supplementary Fig. S1). Interestingly, a previous *in vitro* study has shown that the inhibitor that primarily targets TYK2 altered the development and proliferation of CD4^+^ T cells, suggesting an involvement of TYK2 in T-cell development^37^. Hence, low expression and/or assumingly altered membrane localization of TYK2 due to Arg231Trp mutation in the patients’ cells may give a negative effect on T-cell development. Still, we cannot eliminate an alternative possibility that lymphopenia observed in the patient could be due to the EBV infection^38^.

Several patients displayed either decreased NK cell activity or a reduced number of natural killer T (NKT) cells, suggesting that low NK/NKT-cell activities are correlated with a susceptibility to EBV-associated lymphoproliferative disease^19,20,22–24^. A recent study using mice has suggested a tumor-surveillance function of TYK2 independent of the kinase activity of this protein^39^. The decreased cytotoxic activities of NK and NKT cells in *TYK2*^-/-^ mice are considered to be involved in virus-induced tumor development^40^. Further assessment of the function of NK/NKT cells in patients will be required to gain a better understanding of the pathogenic mechanisms of EBV-associated B-cell lymphoma. Notably, other studies reported finding gain-of-function mutations of *TYK2* in cells derived from a patient with CD30-positive lymphoproliferative disorders^41^ and T-cell acute lymphoblastic leukemia (T-ALL)-derived cell lines^42^. These observations suggest that in T-ALL patients, the survival of aberrant cells is attained through increased STAT1 phosphorylation followed by the upregulation of the anti-apoptotic BCL2 protein^42^. To clarify the tumorigenic mechanism in these patient’s cells, further detailed analysis of the tumor tissue is necessary.

In conclusion, we identified novel compound heterozygous mutations in *TYK2* associated with primary immunodeficiency characterized by a rare type of EBV-associated B-cell lymphoma and T-cell lymphopenia. Complete TYK2 deletion genotypes have been previously reported; however, this is the first report of mutations that are associated with reduced TYK2 expression. The results of cytokine response study using patient’s cells demonstrated partial loss of function of TYK2 which might be involved in the unique clinical phenotypes in the patients. The combination of a rare type of EBV-associated B-cell lymphoma with severe T-cell lymphopenia associated with compound heterozygous *TYK2* mutations represents a distinct disease entity.

## Methods

### Subjects

We investigated two siblings (P1 and P2) from non-consanguineous parents originating from and living in Japan. Both patients were chemotherapy-free at the time of the immunophenotyping. The immunophenotyping was conducted 3 years after the last chemotherapy of P1 that was treated with JPLSG B-NHL03 Group 3^43^ and 4 months after the last chemotherapy of P2 that was treated with FAB/LMB96 Group B^44^. Given that cells from P1, who died from GVHD, were no longer available, immunological characterization was only performed with the cells from P2. P2 was not treated with any immunosuppressive drugs at the time of the experiments. Twice blood collections were required for (A) making growth curves and generation of EBV-BCLs which is used for IFN responsiveness assay and (B) performing IL-6, IL-10, IL-12, IL-23 responsiveness assay. Experiment (A) was conducted 2 years and 10 months after the first chemotherapy with cyclophosphamide, vincristine, prednisolone, methotrexate and cytarabine. Experiment (B) was conducted 6 months after the second chemotherapy with 4 courses of rituximab, etoposide, prednisolone, vincristine, cyclophosphamide and doxorubicin.

### Histologic examination

Biopsies of primary and relapsed parotid lesions and those of primary parotid and nasopharynx lesions were collected from P1 and P2, respectively. Each biopsy was embedded in paraffin for conventional histological and immunohistochemical analyses and was examined for morphological characteristics based on the WHO classification. Immunophenotyping were based primarily on CD20-positive expression. *In situ* hybridization analysis was performed to determine the EBERs and *κ*/*λ* expressions. The INFORM Kappa, Lambda and EBER Probes were purchased from Ventana Medical Systems.

### Mutation screening

Genomic DNA of PBMCs isolated from EDTA-anticoagulated blood was obtained using the QIAamp DNA Blood Mini Kit (QIAGEN). Total RNA was isolated with the RNeasy Plus Mini Kit (QIAGEN), and used to synthesize cDNA with the SuperScript III First-Strand Synthesis System (Life Technologies) and random hexamers, according to the manufacturer’s protocol. Targeted exome sequencing was performed using the genomic DNA isolated from P1, P2 and their parents. We followed the manufacturer’s protocol for the Illumina TruSight Exome Kit to build a genomic library starting from 50 ng of the genomic DNA samples. The enriched libraries were sequenced to obtain 75-bp paired-end reads on an Illumina MiSeq. All the sequence data were aligned to the human reference genome sequence (GRCh37/hg19) with the Burrows-Wheeler Aligner (BWA) program (http://bio-bwa.sourceforge.net). Genetic variants were identified with the Genome Analysis Toolkit (https://software.broadinstitute.org/gatk/), followed by data annotation using the Illumina VariantStudio software. Analyses of the SVs from the exome sequencing data were performed using the programs, Pindel^45^, Delly^46^ and gridss (https://github.com/PapenfussLab/gridss), at their default settings. For Sanger DNA sequencing, coding regions of the *TYK2* gene were amplified from the synthesized cDNA using the following primer set: forward (5′-TTTGAATTCTTGCTTGAGTTGACACAGGGAGCT-3′) and reverse (5′-TTTGCGGCCGCTCTCTAGACAGGAGTAAGGCACAC-3′). The resulting DNA fragment was cloned into the pCR-Blunt II TOPO vector (Life Technologies).

### Antibodies and cytokines

The anti-CD19 and anti-CD27 mAbs were purchased from Sigma-Aldrich and the anti-CD20 mAb from Ventana Medical Systems. The antibodies against human TYK2, STAT1, STAT3 and STAT4, and their phosphorylated forms were purchased from Cell Signaling Technology. The antibodies directed against ß-tubulin, STAT2, phosphorylated STAT2 and horseradish peroxidase-conjugated rabbit polyclonal antibody were obtained from Abcam. The human IL-2, IL-12 and IL-23 recombinant proteins were purchased from Cell Signaling Technology, while IFN-α was obtained from Sigma-Aldrich, and IFN-ß, IL-6 and IL-10 were purchased from Peprotech.

### Cell culture and stimulation

CD4^+^ T cells were prepared from the heparinized blood of patients and healthy donors as previously described^47^. Before stimulation with cytokines, the expanded CD4^+^ T cells were washed and serum-starved for 18 h in RPMI-1640 supplemented with 1% FBS. The cells were then stimulated with IFN-α (1000 U/mL), IFN-ß (500 U/mL), IL-6 (10 ng/mL), IL-10 (10 ng/mL), IL-12 (10 U/mL) or IL-23 (500 ng/mL) at 37 °C for 15 min.

### Western blot analysis

After stimulation with cytokines, cells were harvested and lysed with lysis buffer [50 mM Tris (pH 8.0), 1% Triton X-100, 150 mM NaCl, 2 mM EDTA, 2 μg/mL aprotinin, 1 mM sodium orthovanadate, 100 μg/mL PMSF and 1 mM NaF], followed by sonication. The protein samples were subjected to 10% SDS-PAGE and transferred onto PVDF membranes (Millipore). The membranes were immunoblotted with primary antibodies followed by the appropriate secondary antibodies. The intensity of the immunoblotted bands was quantified using ImageQuant (GE Healthcare).

### Gene expression analysis

For the analysis of IFN-inducible genes, EBV-BCLs and freshly isolated PBMCs were stimulated with IFN-α (1000 U/mL) or IFN-ß (500 U/mL) for 2 h. Quantification of the cDNA generated by reverse transcription from total RNA was performed using a real-time PCR assay in a Thermal Cycler Dice Real-Time System TP800 (Takara Bio) according to the manufacturer’s protocol for SYBR Premix DimerEraser (Takara Bio). Real-time PCR assays were employed to analyze the levels of *cyclophilin B*, *TYK2*, *IRF1*, *SOCS1*, *SOCS3*, *STAT1* and *TAP1* mRNA using each primer set listed in Supplementary Table S3. The expression level of each gene was determined using the standard curve method.

### Statistics

All data are expressed as mean ± SEM. Statistical significance was determined using 2-tailed Student’s t-test. For all studies, values of P < 0.05 were considered statistically significant.

### Study approval

Written informed consent for the genetic and functional investigations was obtained from the patients and their parents in accordance with local regulations, with approval by the ethics committee of Nagoya Medical Center (registration #2013-679). The experiments were conducted at the Clinical Research Center, National Hospital Organization Nagoya Medical Center, Japan.

### Data Availability

Exome sequencing data were deposited in the Japanese Genotype-phenotype Archive (JGA)^48^ under accession number JGAS00000000098 under Type I security.

## Electronic supplementary material

Supplementary Figures
