# Supplementary material for: Compound heterozygous TYK2 mutations underlie primary immunodeficiency with T-cell lymphopenia
Source: Sci Rep. 2018 May 3;8:6956. doi: 10.1038/s41598-018-25260-8 (PMC5934390; doi:10.1038/s41598-018-25260-8)
Supplement: Supplementary file 1 — Supplementary Figures [file 41598_2018_25260_MOESM1_ESM.pdf]

Compound heterozygous TYK2 mutations underlie primary immunodeficiency with T-cell lymphopenia

Michiko Nemoto[1,2], Hiroyoshi Hattori[1,3]*, Naoko Maeda[3], Nobuhiro Akita[3], Hideki Muramatsu[4], Suzuko Moritani[5], Tomonori Kawasaki[5], Masami Maejima[1] Hirotaka Ode[1], Atsuko Hachiya[1], Wataru Sugiura[1], Yoshiyuki Yokomaku[1], Keizo Horibe[1,3] and Yasumasa Iwatani[1, 6]*

1Clinical Research Center, National Hospital Organization Nagoya Medical Center, Aichi 460-0001, Japan;
2Graduate School of Environmental and Life Science, Okayama University, Okayama 700-8530, Japan;
3Department of Pediatrics, National Hospital Organization Nagoya Medical Center, Aichi 460-0001, Japan;
4Department of Pediatrics, Nagoya University Graduate School of Medicine, Aichi 466-8550, Japan;
5Department of Pathology, National Hospital Organization Nagoya Medical Center, Aichi 460-0001, Japan;
6Division of Basic Medicine, Nagoya University Graduate School of Medicine, Aichi 466-8550, Japan,

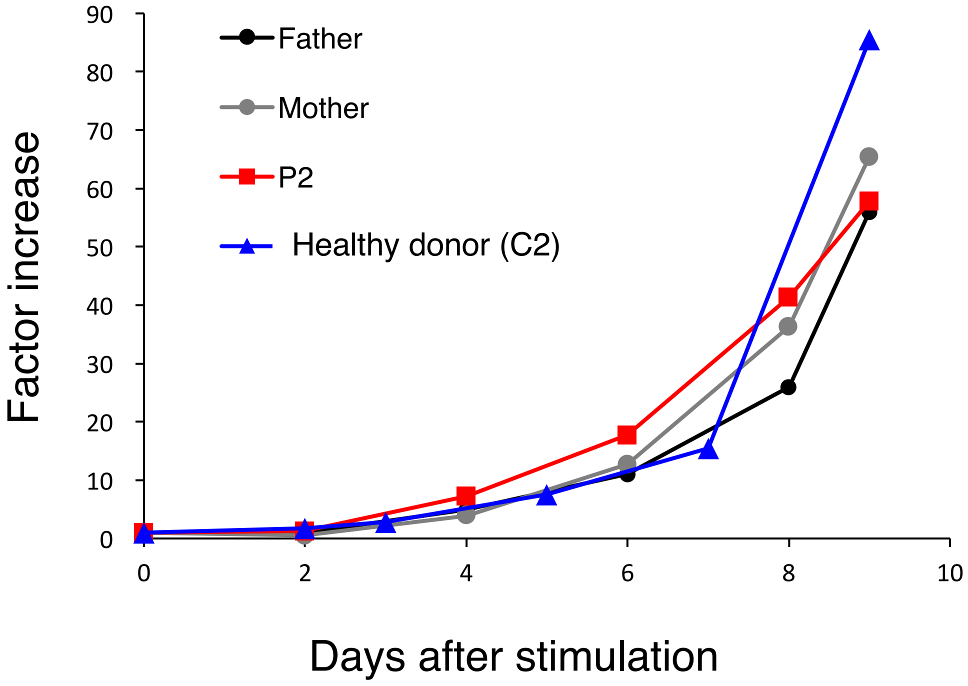

Supplementary Figure S1
Growth curves of T cells stimulated with anti-CD3 plus anti-CD28 coated beads and expanded in culture with IL-2.

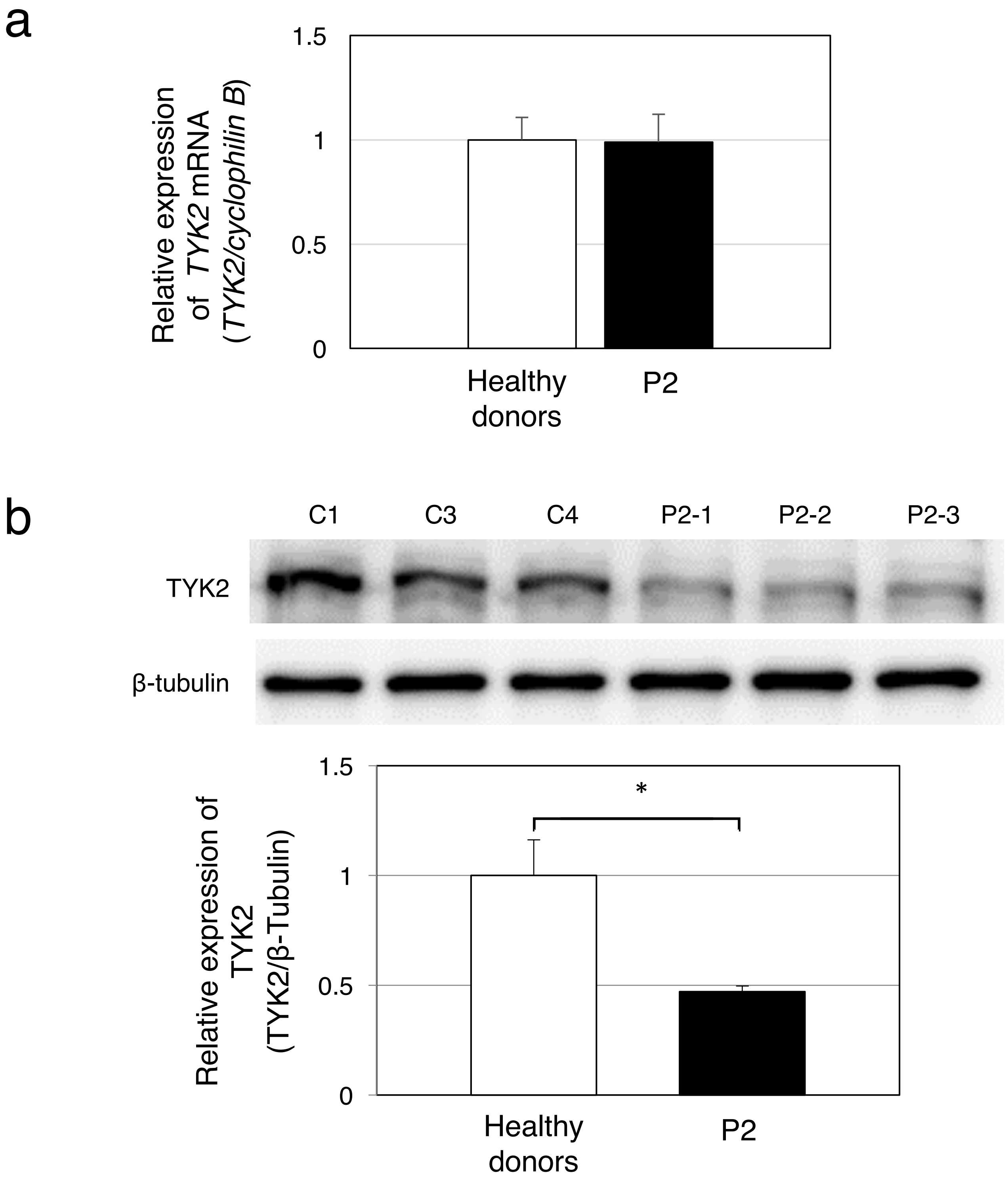

Supplementary Figure S2

Expression Levels of TYK2 mRNA and protein in EBV-BCLs. (a) *TYK2* mRNA expression in EBV-BCLs from healthy donors (C1, C3, C4) and a *TYK2*-mutated patient (P2). (b) Immunoblots for TYK2 expression in EBV-BCLs from healthy donors (C1, C3, C4) and a *TYK2*-mutated patient (P2). P2-1, P2-2, and P2-3 are different batches of EBV-BCLs from the same patient. β-tubulin serves as a loading control.

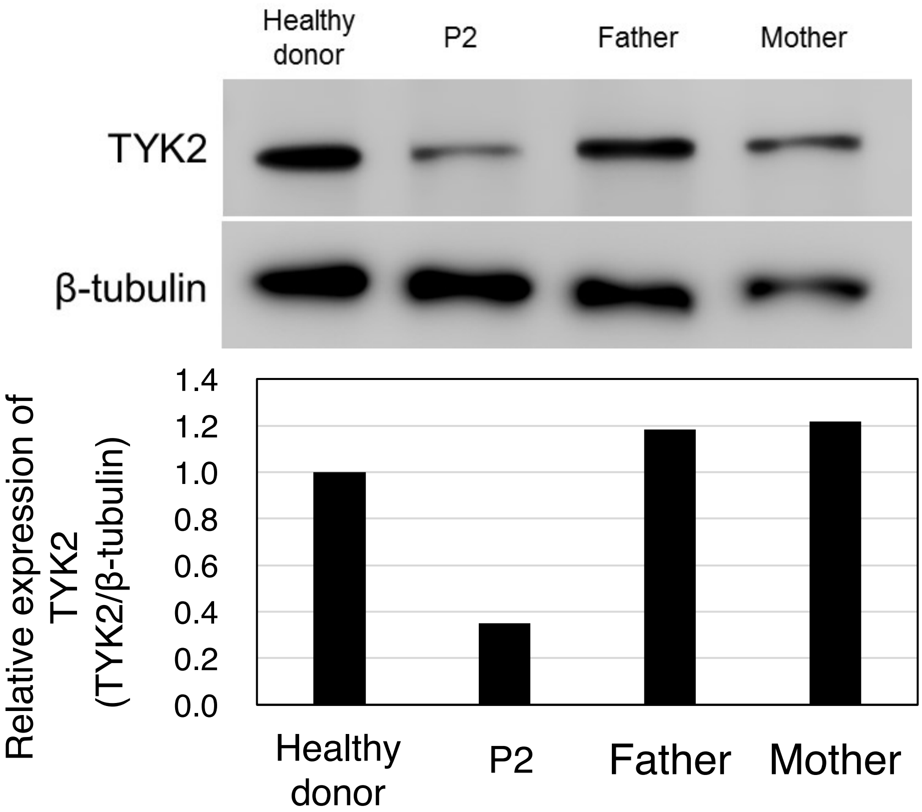

Supplementary Figure S3

Expression Levels of TYK2 protein in $CD4^+$ T cells . Immunoblots for TYK2 expression in $CD4^+$ T cells from a healthy donor (C1), a TYK2-mutated patient and her parents. β-tubulin serves as a loading control.

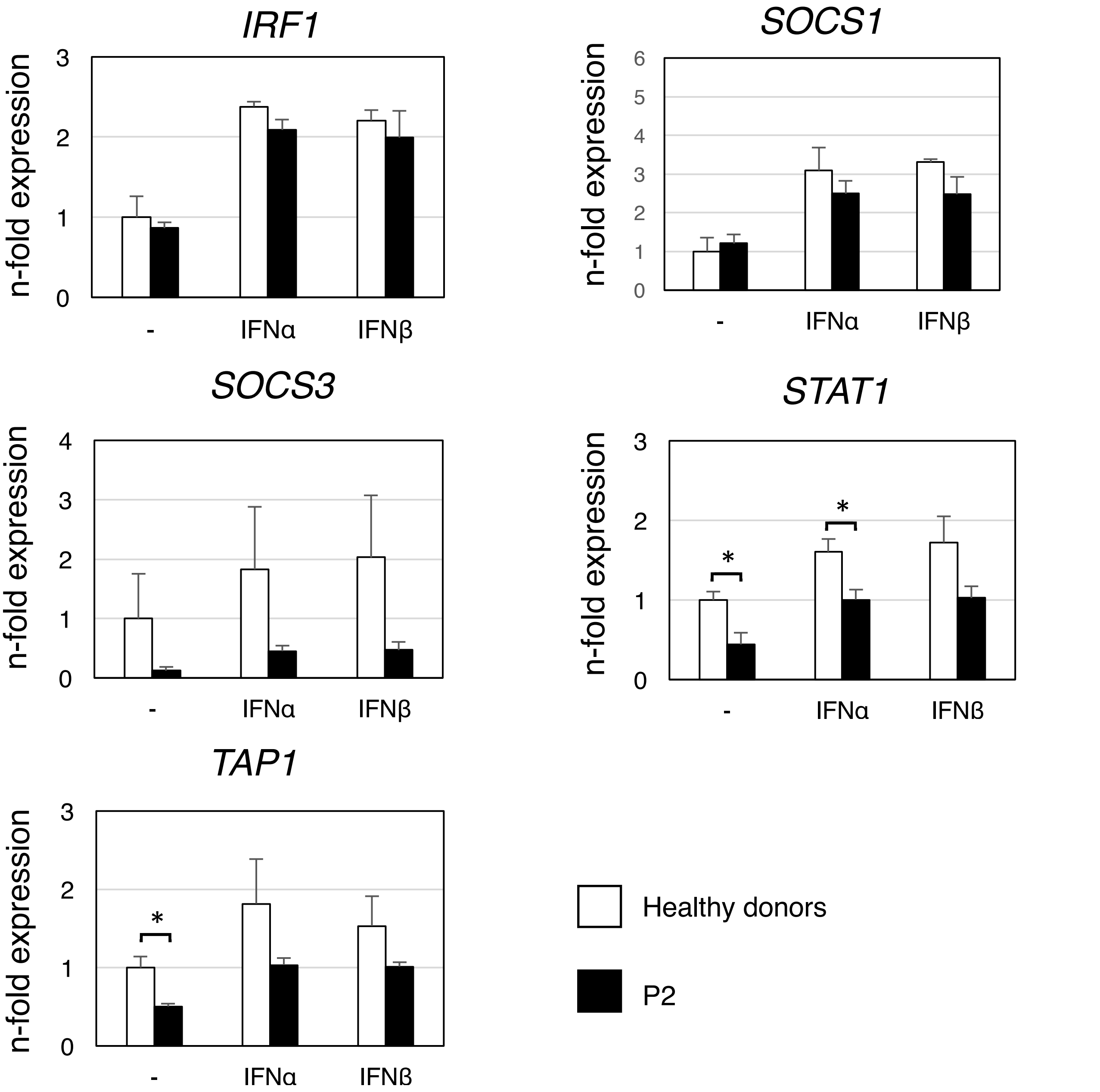

Supplementary Figure S4
Analysis of type I IFN-inducible gene induction in EBV-BCLs from healthy donors (C1, C3, C4) and a TYK2-mutated patient. Cells were stimulated with IFNα (1000 U/mL), IFNβ (500 U/mL) or without any IFN for 2 h. The cDNAs generated from the total RNA were quantified with qPCR assays. The expression levels of *IRF1*, *SOCS1*, *SOCS3*, *STAT1* and *TAP1* mRNAs were determined by normalizing each with *cyclophilin B* levels. The induction level is presented as n-fold expression that in the untreated healthy donor cell control, which was set as 1. The data are derived from three independent experiments using 3 EBV-BCLs per genotype. Data represent mean ± SEM. *$P < 0.05$. P values were derived from 2-tailed Student's t-test.

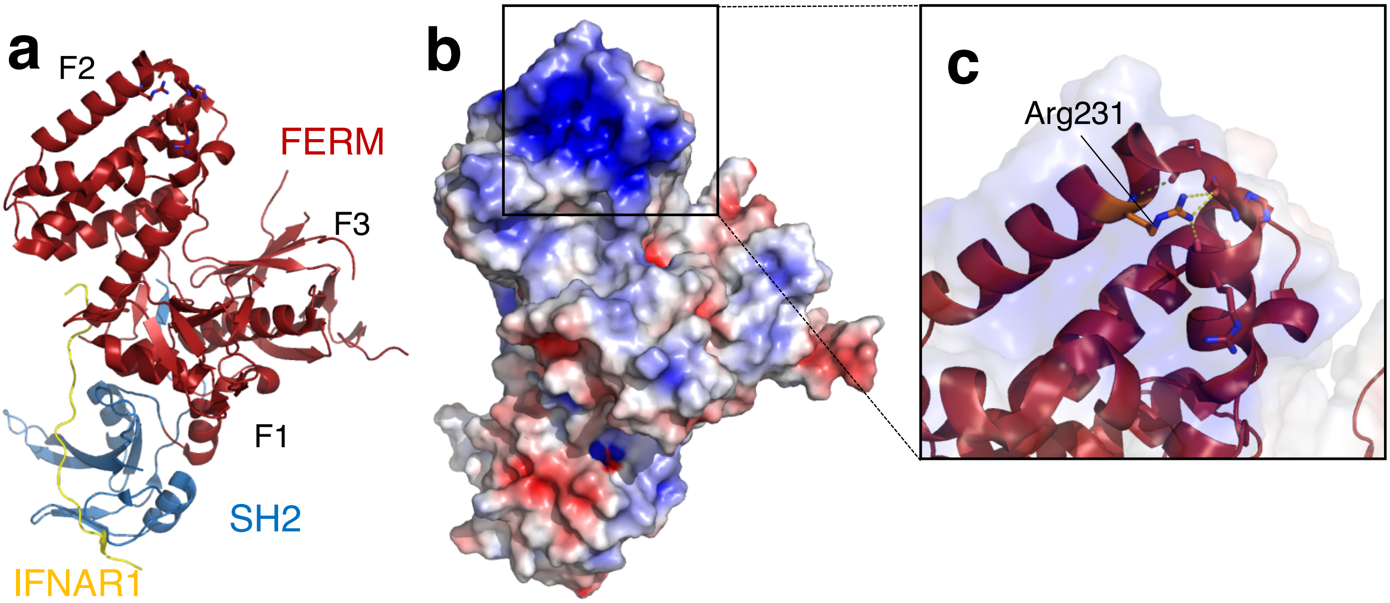

Supplementary Figure S5

Mapping of the Arg231 in the crystal structure of human TYK2 structure (PDB 4PO6). (a) Ribbon representation of the structure of the FERM and SH2 domains is in red and blue, respectively. IFN-α/ß receptor subunit 1 (IFNAR1) interacts with the FERM F2 and SH2. (b) Electrostatic surface potential, contoured at -5 (red) to +5 (blue) *kT/e*, is shown for the complex. The FERM F2 contains a highly basic patch, which likely adheres to the plasma membrane. (c) The detailed view around Arg231 (orange) is shown. The Arg231 constitutes the basic patch and likely stabilizes the F2 conformation by forming intramolecule hydrogen bonds. The Arg231Trp mutation may lead to disruption of the positively charged-F2 conformation, which shows defects in function.

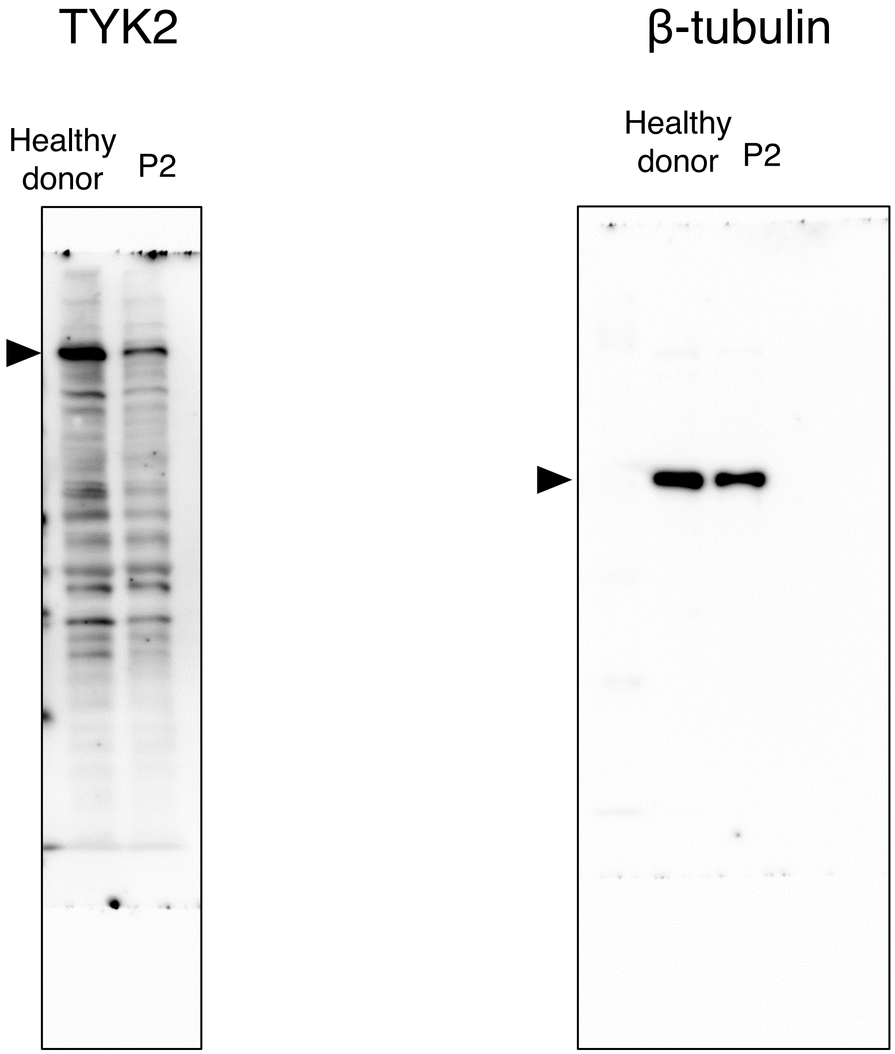

Supplementary Figure S6
Full-length immunoblot images of Figure1d.
Each arrow head highlights bands for TYK2 or β-tubulin.

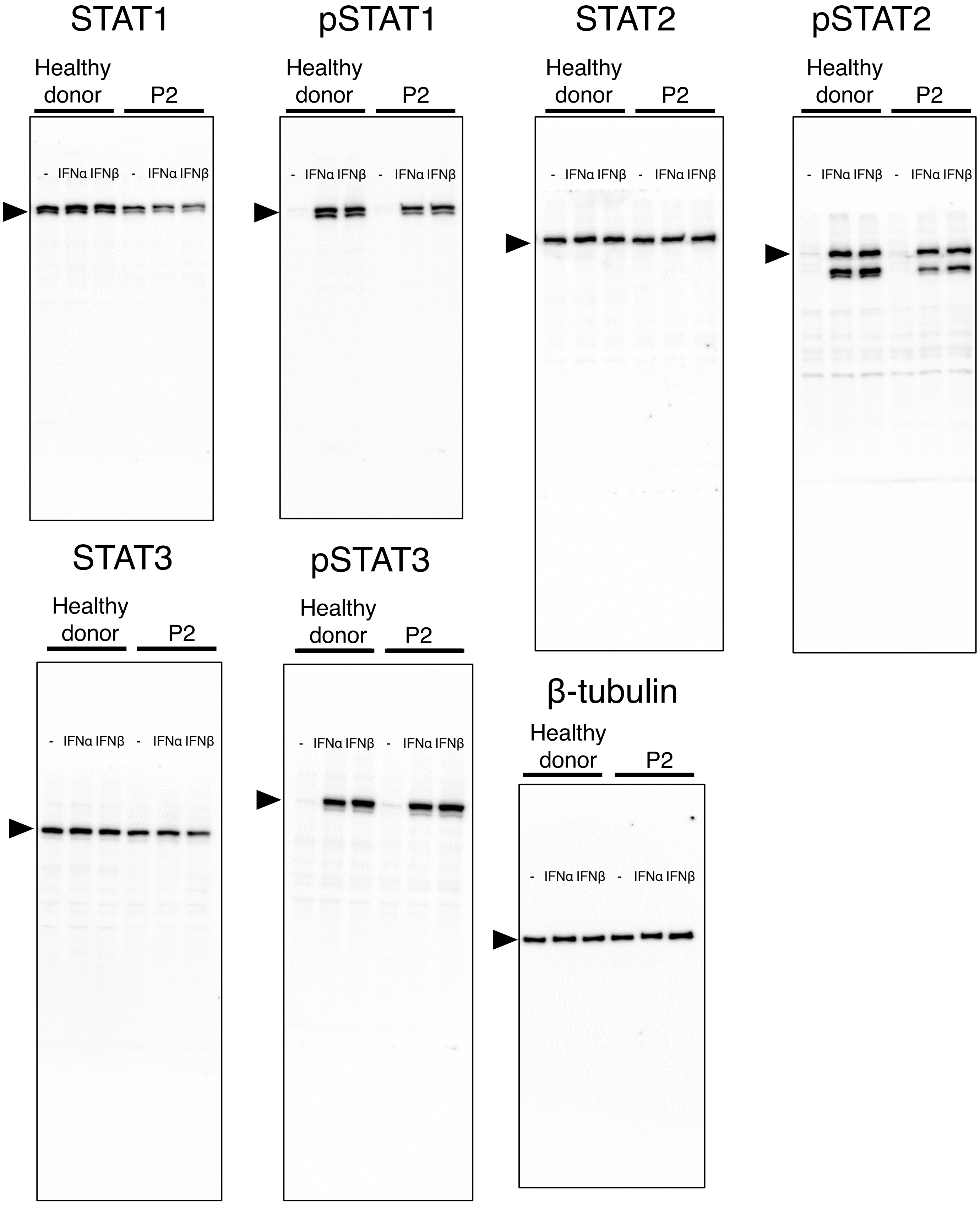

Supplementary Figure S7

Full-length immunoblot images of Figure3a. Each arrow head highlights bands for STAT1, pSTAT1, STAT2, pSTAT2, STAT3, pSTAT3, or β-tubulin.

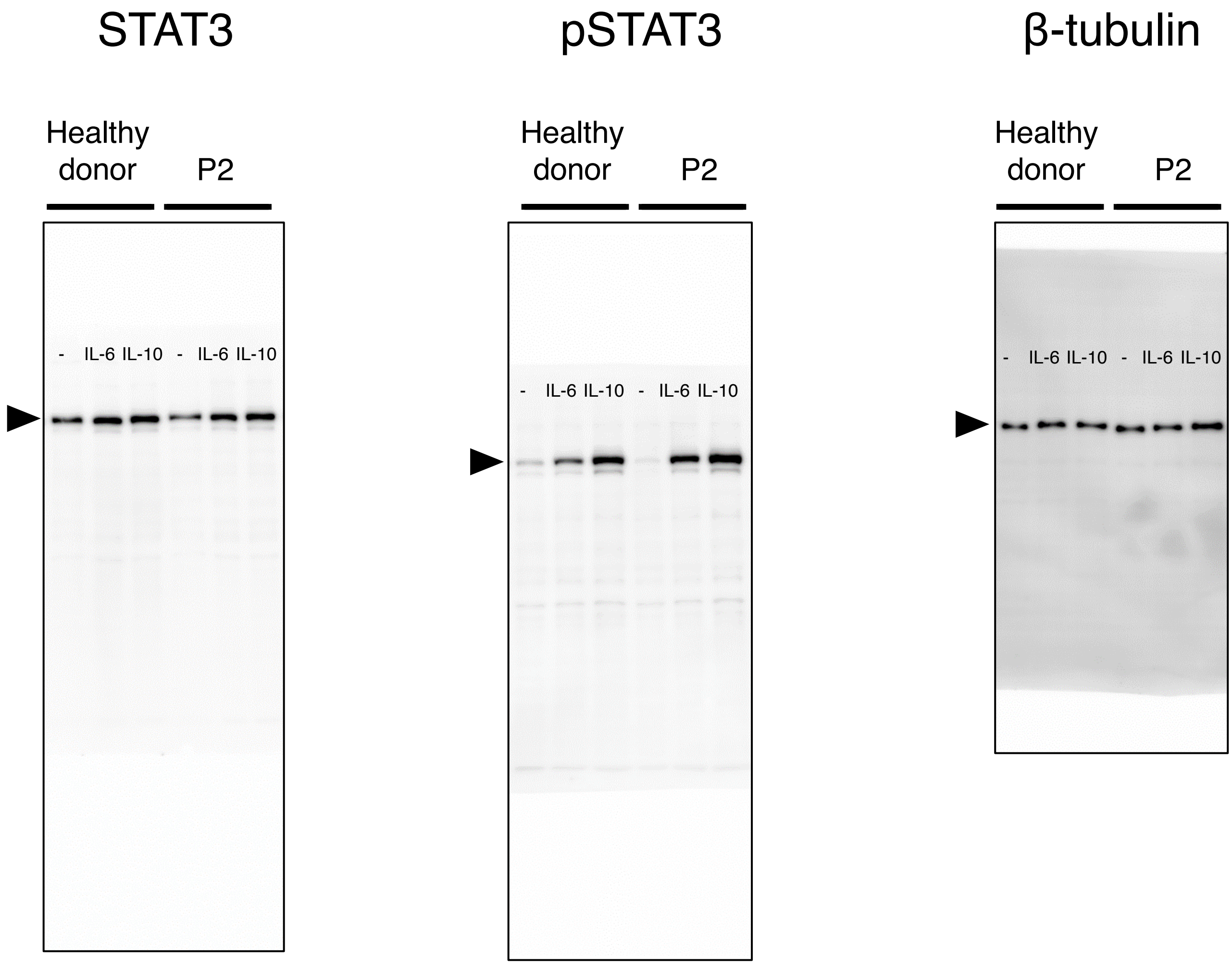

Supplementary Figure S8
Full-length immunoblot images of Figure5a.
Each arrow head highlights bands for STAT3, pSTAT3, or β-tubulin.

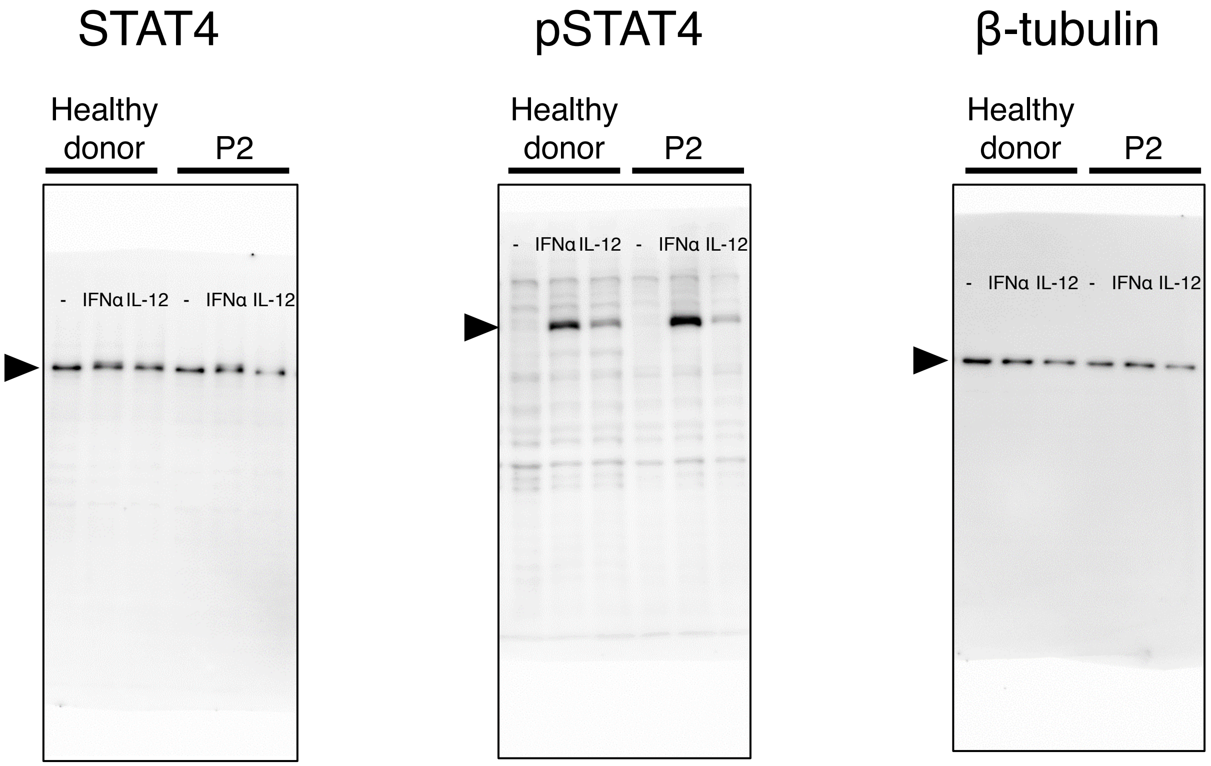

Supplementary Figure S9
Full-length immunoblot images of Figure5b.
Each arrow head highlights bands for STAT4, pSTAT4, or β-tubulin.

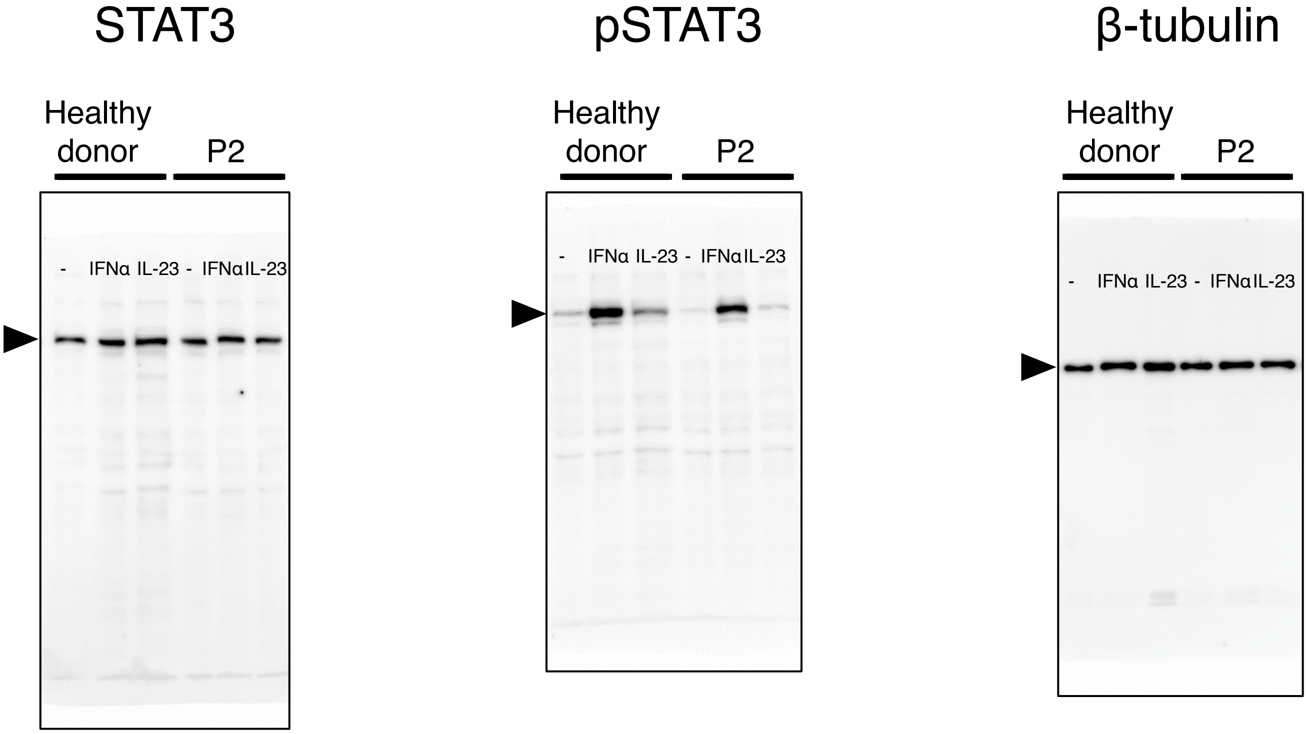

Supplementary Figure S10
Full-length immunoblot images of Figure5c.
Each arrow head highlights bands for STAT3, pSTAT3, or β-tubulin.
